# Metabolic Reprogramming in Stromal and Immune Cells in Rheumatoid Arthritis and Osteoarthritis: Therapeutic Possibilities

**DOI:** 10.1002/eji.202451381

**Published:** 2025-04-01

**Authors:** Órlaith C. Henry, Luke A. J. O'Neill

**Affiliations:** ^1^ Biomedical Sciences Institute Trinity College Dublin Dublin Ireland

**Keywords:** arthritis, inflammation, metabolic reprogramming, metabolism, rheumatology

## Abstract

Metabolic reprogramming of stromal cells, including fibroblast‐like synoviocytes (FLS) and chondrocytes, as well as osteoclasts (OCs), are involved in the inflammatory and degenerative processes underlying rheumatoid arthritis (RA) and osteoarthritis (OA). In RA, FLS exhibit mTOR activation, enhanced glycolysis and reduced oxidative phosphorylation, fuelling inflammation, angiogenesis, and cartilage degradation. In OA, chondrocytes undergo metabolic rewiring, characterised by mTOR and NF‐κB activation, mitochondrial dysfunction, and increased glycolysis, which promotes matrix metalloproteinase production, extracellular matrix (ECM) degradation, and angiogenesis. Macrophage‐derived immunometabolites, including succinate and itaconate further modulate stromal cell function, acting as signalling molecules that modulate inflammatory and catabolic processes. Succinate promotes inflammation whilst itaconate is anti‐inflammatory, suppressing inflammatory joint disease in models. Itaconate deficiency also correlates inversely with disease severity in RA in humans. Emerging evidence highlights the potential of targeting metabolic processes as promising therapeutic strategies for connective tissue disorders.

## Graphical abstract

Immune cells and stromal cells undergo metabolic reprogramming in both rheumatoid and osteoarthritis. This review summarises current evidence on the metabolic changes within and the crosstalk between immune and stromal cells in these diseases. In addition, we explore the therapeutic potential of targeting metabolic processes for the treatment of arthritis.

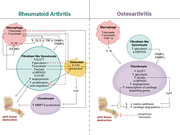

**Abbreviations**
4‐OI: 4‐octyl itaconate
Abat: 4‐aminobutyrate aminotransferase
ACOD1: aconitate decarboxylase 1
Akt: protein kinase B
AMPK: adenosine monophosphate‐activated protein kinase
AMPKα: adenosine monophosphate‐activated protein kinase alpha
ATP: adenosine triphosphate
CIA: collagen‐induced arthritis
DAMP: damage‐associated molecular pattern
DMARD: disease‐modifying antirheumatic drugs
DMM: dimethyl malonate
ECM: extracellular matrix
ERK1: extracellular signal‐regulated kinase 1
FGF: fibroblast growth factor
FLS: fibroblast‐like synoviocytes
GC: glucocorticoid
GLUT1: glucose transporter 1
GPCR: g‐protein‐coupled receptor
GPX4: Glutathione peroxidase 4
GSK‐3β: glycogen synthase kinase 3 beta
HIF1‐α: hypoxia‐inducible factor 1‐alpha
HK2: hexokinase 2
HSP90: heat shock protein 90
IFN‐γ: interferon gamma
IL‐1β: interleukin‐1 beta
IL‐6: interleukin‐6
IRG1: immunoresponsive gene 1
LPS: lipopolysaccharide
MAPK: Mitogen‐activated protein kinase
MMP: matrix metalloproteinase
MSC: mesenchymal stromal cell
mtDNA: mitochondrial DNA
mTOR: mammalian target of rapamycin
NADPH: nicotinamide Adenine Dinucleotide Phosphate Hydrogen
NF‐κB: nuclear factor‐kappa B
NLRP3: nucleotide‐binding domain, leucine‐rich‐containing family, pyrin domain‐containing‐3
NRF1: nuclear respiratory factor 1
NRF2: nuclear factor erythroid 2‐related factor 2
OA: osteoarthritis
OC: osteoclast
OCR: oxygen consumption rate
OXPHOS: oxidative phosphorylation
PAMP: pathogen‐associated molecular pattern
PBMC: peripheral blood mononuclear cell
PDH1: pyruvate dehydrogenase 1
PDK3: pyruvate dehydrogenase lipoamide kinase isozyme 3
PGK1: phosphoglycerate kinase 1
PI3K: phosphoinositide 3‐kinase
PKM2: pyruvate kinase isozymes M2
PRR: pathogen recognition receptor
RA: rheumatoid arthritis
ROS: reactive oxygen species
SDH: succinate dehydrogenase
siRNA: small interfering ribonucleic acid
SIRT: sirtuin 1
STING: stimulator of interferon genes
SUCNR1: succinate receptor 1
TCA: tricarboxylic acid
TFAM: mitochondrial transcription factor A
TLR: Toll‐like receptor
TNF‐α: tumour necrosis factor‐alpha
VEGF: vascular endothelial growth factor

## 1 Introduction

Stromal cells within tissues are as fundamental as immune cells to the pathogenesis of chronic inflammatory and autoimmune diseases, often shaping the specific characteristics of each disease based on the tissue under attack [1]. For example, synovium and synovial inflammation are central to the pathogenesis of rheumatoid arthritis (RA) and osteoarthritis (OA), and fibroblasts and chondrocytes serve as key players in these diseases [2, 3, 4, 5, 6, 7, 8]. Studies from the past couple of decades have investigated the activation of stromal cells in response to signals such as cytokines and Toll‐like receptors (TLRs). There has also been a focus on metabolic changes in immune cells that shape their phenotype, but less attention has been paid to metabolic changes in stromal cells. The field of immunometabolism has revealed unexpected new roles for metabolites in promoting immunity and inflammation. There is now evidence that the metabolic reprogramming of stromal cells could underpin RA and OA.

Despite advances in treatment options and the availability of effective disease‐modifying antirheumatic drugs (DMARDs), many RA patients still fail to achieve remission [9]. This unmet need, compounded by the vulnerability of patients to infections, and the high cost of DMARDs, underscores the necessity for new RA treatments. Additionally, no cure currently exists for OA due to the challenges of cartilage regeneration after damage. This review will focus on the existing knowledge on metabolic reprogramming of fibroblast‐like synoviocytes (FLS), chondrocytes, and osteoclasts (OCs) in rheumatoid and osteoarthritis, how macrophage metabolites are utilised as signalling molecules to communicate with these cells and the potential that exists for manipulating immune and non‐immune cell metabolism for developing therapeutics for these diseases.

## 2 Metabolic Reprogramming in FLS

Rheumatoid arthritis involves FLS being stimulated either by endogenous ligands of pattern recognition receptors (PRRs) or by products of immune cells such as macrophages, neutrophils, and lymphocytes [10]. As a result, the FLS undergo differentiation into two functionally distinct subtypes that exhibit either proinflammatory or tissue‐degrading properties. The pro‐inflammatory phenotype is defined by the expression of thymus cell antigen 1 (THY1, CD90) [11, 12]. The activated FLS contribute to the formation of the pannus, an abnormal tissue growth in the joint, which functions as a tertiary lymphoid organ by engaging with immune cells [10].

FLS, like stromal cells from other tissues, can recognise pathogen‐associated molecular patterns (PAMPs) and damage‐associated molecular patterns (DAMPs) via their PRRs. They express TLR1‐6, with TLR3 and TLR4 being most abundant [13]. The TLR4 pathway can be activated by citrullinated histones and fibrinogen, or the ECM protein tenascin C, leading to RA pathogenesis [14, 15, 16]. Macrophages, potentially recruited to the joint by chemokines released from FLS, can further activate FLS by producing pro‐inflammatory cytokines, chemokines, and growth factors, inducing the FLS to produce IL‐6, prostanoids, and matrix metalloproteinases (MMPs) which create a vicious network that perpetuates synovial inflammation [17]. Whether FLS are a dominant source of chemokines is however not known.

T and B lymphocytes also interact bidirectionally with the FLS to drive RA [10]. FLS‐derived cytokines and chemokines aid in the recruitment and differentiation of T cells to the joint, and IL‐17A produced by Th17 cells activates FLS. FLS also produce significant amounts of B‐cell‐activating factor and a proliferation‐inducing ligand as well as IL‐6 which affects B cell functions. Activated FLS therefore display increased proliferation, invasion, and migration, while also producing cytokines and chemokines that exacerbate the inflammatory milieu within the arthritic joint. To enact these functional changes, a metabolic shift in the FLS is essential.

In FLS derived from the inflamed tissue of RA patients, there is increased glycolysis, and glycolytic inhibitors have been shown to reduce the severity of inflammatory arthritis symptoms in murine models [18]. This increase in FLS glycolysis found in RA results in increased lactate and succinate production. Increased extracellular lactate can contribute to RA pathogenesis by stimulating fibroblast proliferation and invasion [19]. Lactate promotes fibroblast growth factor (FGF) production by osteoblasts via the NF‐κB signalling pathway [19]. FGF can in turn induce chondrocytes to produce MMP13 via activation of the PI3K/Akt and ERK1/2 pathways, ultimately leading to exacerbated tissue destruction in the RA joint [20].

Activation of the PI3K/Akt signalling pathway can also result in the downstream activation of GSK‐3β, a serine/threonine protein kinase, facilitating the subsequent phosphorylation of mTOR. Activated mTOR drives Akt activation and promotes the upregulation of the glucose transporter GLUT1, as well as the expression of HIF1‐α, culminating in increased angiogenesis and glycolysis [21].

Kwon et al. investigated the effects of administering GSK‐3β inhibitors in collagen‐induced arthritis (CIA) in rats, finding that these inhibitors significantly reduced arthritic symptom severity, as well as serum levels of IL‐1β, IL‐6, and TNF‐α. These improvements were accompanied by notable metabolic alterations, characterised by decreased levels of NF‐κB, JNK, c‐Jun, ATF2, and p38 [22]. Additionally, both lactate and succinate can stabilise HIF‐1α in macrophages which results in the production of pro‐inflammatory cytokines such as IL‐1β [23, 24]. This further contributes to the inflammatory environment in the RA joint.

A number of studies have demonstrated increased synovial HIF‐1α expression in RA [25, 26]. Moreover, HIF‐1α expression has been observed in both the synovium of animal models and in the monocytes of individuals at risk of RA development [27, 28].

**FIGURE 1:**
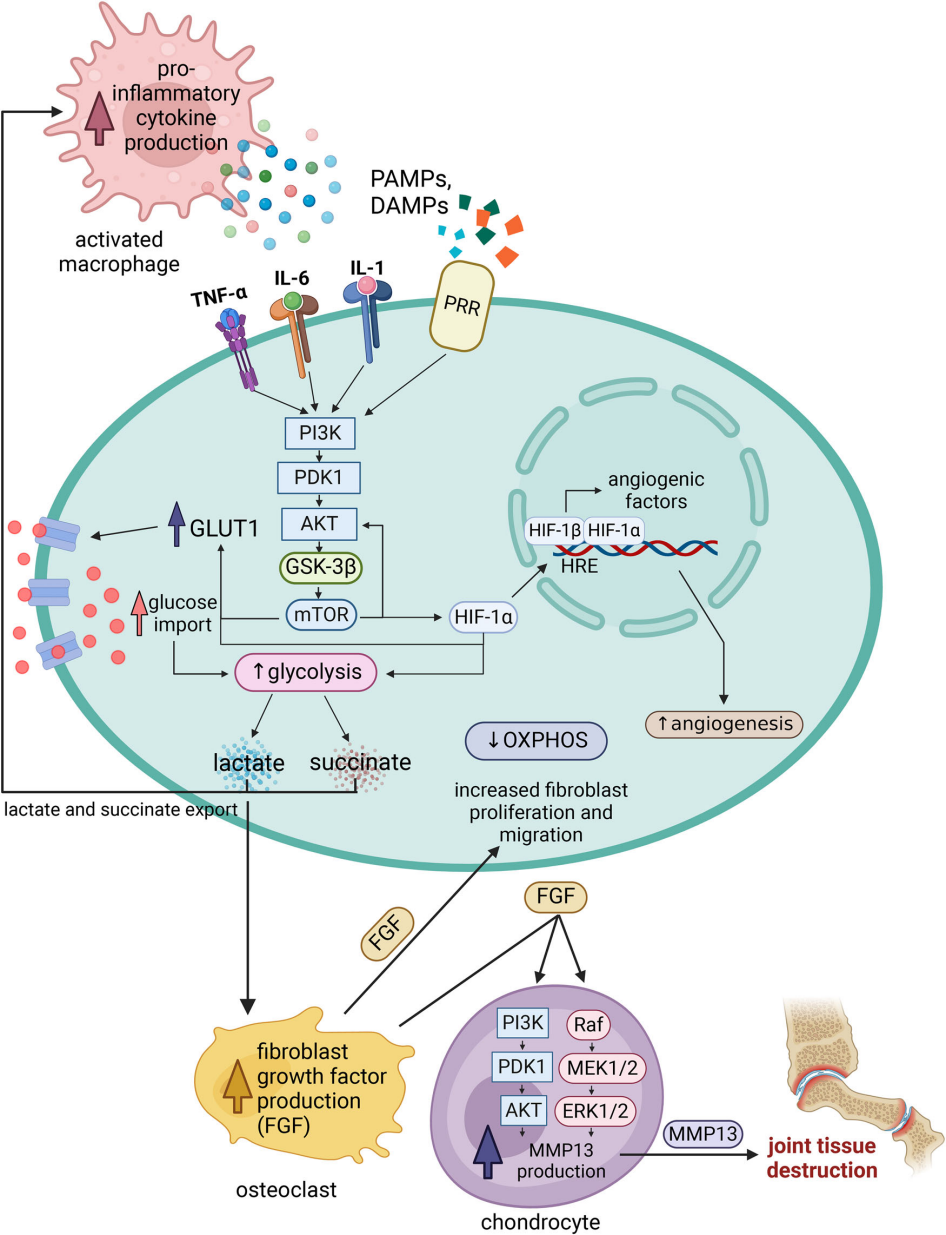
Metabolic Reprogramming in rheumatoid arthritis (RA) fibroblast‐like synoviocytes (FLS). In RA, FLS play a central role in driving inflammation and joint destruction. They are stimulated either by PRR ligands called PAMPs and DAMPs, or by immune cell‐derived cytokines such as TNF, IL‐6, and IL‐1. This stimulation causes activation of the PI3K/Akt/mTOR pathway, leading to metabolic reprogramming in FLS. mTOR can cause upregulation of the glucose transporter GLUT1, driving increased glucose import which subsequently fuels a shift towards aerobic glycolysis (Warburg‐like metabolism), ultimately resulting in elevated lactate and succinate levels. These metabolites act as signalling molecules that further stimulate immune cells, such as macrophages, to produce pro‐inflammatory cytokines such as TNF, IL‐6, and IL‐1, perpetuating a feedback loop that sustains FLS activation. Alongside inflammation, metabolic dysregulation in RA FLS also impacts joint destruction. Both itaconate and succinate can stimulate OCs to produce FGF, which drives FLS proliferation and migration, contributing to synovial hyperplasia, pannus formation, and cartilage degradation, driving RA pathogenesis. Additionally, FGF can drive MMP13 production, an enzyme responsible for the degradation of ECM components, promoting joint tissue destruction. Mechanistically, these effects have been shown to occur through the PI3K/PDK1/AKT and Raf/MEK/ERK pathways. Apart from its role in driving increased glycolysis, mTOR can also stabilise HIF‐1α, a key transcription factor that promotes angiogenesis. HIF‐1α translocates to the nucleus where it facilitates transcription of pro‐angiogenic factors that contribute to increased blood vessel formation in the inflamed synovium. This vascularisation facilitates further immune cell infiltration and nutrient supply to the expanding synovium, enhancing disease progression. PAMPs—pathogen‐associated molecular patterns; DAMPs—damage‐associated molecular patterns; TNF‐α—tumour necrosis factor‐alpha; IL‐6–interleukin 6; IL‐1—interleukin 1; PRR—pattern recognition receptor; PI3K—phosphoinositide 3‐kinase; PDK1—pyruvate dehydrogenase kinase 1; AKT—Protein kinase B; GSK‐3β—glycogen synthase kinase 3 beta; mTOR—mitochondrial target of rapamycin; GLUT1–glucose transporter 1; HIF‐1α—hypoxia‐inducible factor 1‐alpha; HIF‐1β—hypoxia‐inducible factor 1‐beta; HRE—hypoxia response elements; OXPHOS—oxidative phosphorylation; FGF—fibroblast growth factor; MEK1/2—mitogen‐activated protein kinase kinase 1 and 2; ERK1/2—extracellular signal‐regulated kinases 1 and 2; MMP13—matrix metalloproteinase 13. Created in https://BioRender.com.

The dysregulation of phosphoglycerate kinase 1 (PGK1) has also been implicated in RA, with heightened expression noted in RA synovial tissue and serum [29]. Administration of PGK1 siRNA markedly reduced both the proliferation and migration of cultured RA FLS. Additionally, reduced levels of IL‐1β and IFN‐γ were detected in the supernatants of these cells. Glutamine, phosphatidylcholine, and amino acid metabolism were also found to be altered in RA FLS [21]. The metabolic changes in FLS are illustrated in Figure 1.

FLS in OA also experience metabolic reprogramming, but metabolomic analyses, including principle component analysis and hierarchical cluster analysis, reveal that RA FLS exhibit a distinctly different metabolic profile compared with OA FLS [30]. The authors identified significant differences in metabolites such as lactose, fructose and mannose between RA FLS and OA FLS. Moreover, due to disturbed amino acid metabolism the levels of amino acids such as isoleucine, leucine, histidine, valine, ornithine, lysine, and others, which serve as a critical energy source under glucose‐deficient conditions were found to be elevated in OA FLS relative to RA FLS. Pathways involved in generating precursors for nucleotide synthesis, glycolysis, and the pentose phosphate pathway were observed to be upregulated in FLS in RA compared with OA, indicative of more rapidly dividing and more invasive FLS in RA compared with OA.

Proteomic analyses and pathway assessments have highlighted elevated pyruvate dehydrogenase kinase 3 (PDK3) expression as a defining feature of proinflammatory THY1^+^ FLS from OA patients [31]. PDK3 is known to phosphorylate pyruvate dehydrogenase 1 (PDH1), resulting in its inactivation, and consequently reducing pyruvate entry into the tricarboxylic acid (TCA) cycle, thereby limiting mitochondrial oxidative phosphorylation of pyruvate, boosting lactate production [32].

Interestingly, OA FLS exhibit a lower basal rate of total respiration, non‐mitochondrial respiration, proton leak, and lower mitochondrial ATP production compared with mesenchymal stromal cells (MSCs) [31]. Nonetheless, despite the lower baseline rate of oxygen consumption, they exhibited a significantly higher amount of ATP per cell relative to MSCs. This was attributed to the PDK3 upregulation in the FLS, which via the inactivation of PDH1 and subsequent redirection of pyruvate to lactate production, causing NAD^+^ regeneration, facilitates rapid glycolysis‐derived ATP production. Moreover, the inhibition of PDKs has been shown to induce a metabolic shift in the FLS from glycolysis to OXPHOS, which was accompanied by diminished cellular proliferation and reduced pro‐inflammatory cytokine and chemokine release.

## 3 Metabolic Reprogramming in Chondrocytes

Although cartilage is sparsely populated with cells, it is very metabolically active. Its limited oxygen and glucose supply compared with other tissues presents chondrocytes with a unique set of metabolic challenges [33]. Recent findings indicate that metabolism is essential for maintaining cartilage homeostasis, and OA chondrocytes undergo a metabolic shift from OXPHOS towards glycolysis [34, 35].

Upon activation by inflammatory stimuli, such as PAMPs, DAMPs, or pro‐inflammatory cytokines like IL‐1β, chondrocytes undergo a metabolic transition from a resting state to an activated state characterised by enhanced glycolysis driven by low‐grade inflammation‐induced hypoxia [36, 37, 38]. This activation facilitates increased glucose uptake and utilisation, enabling quicker ATP production for the rapidly proliferating and invading cells in OA [39]. The oxygen‐deficient microenvironment further stimulates the upregulation of GLUT1 expression [40]. Studies have attributed increased advanced glycation end products which play a role in cartilage degradation to an upregulation of GLUT1 expression [41]. Moreover, GLUT1 levels have also been shown to increase with OA disease severity [42].

In OA, there is a notable increase in the gene expression of hexokinase 2 (HK2), the first enzyme in the glycolytic pathway, alongside elevated levels of its product glucose‐6‐phosphate. This suggests a significant upregulation of glycolysis. Another glycolytic enzyme, pyruvate kinase M2 (PKM2) was shown to be upregulated in OA chondrocytes [43]. This upregulation of PKM2 expression causes an accumulation of lactate which decreases the pH of the microenvironment. This acidic milieu adversely affects the matrix synthesis capabilities of chondrocytes and may promote their degradation of the cartilage [44, 45]. Adenosine monophosphate‐activated protein kinase (AMPK), an essential regulator of homeostasis, exhibits diminished activity in OA [46]. In a murine model featuring cartilage‐specific, tamoxifen‐inducible knockout of AMPKα, the deletion of AMPKα in adulthood was associated with accelerated progression of OA [47]. The osteoarthritic joint is characterised by an abundance of inflammatory cytokines which can ultimately activate the chondrocytes [48]. NF‐κB activation in chondrocytes is believed to enhance the expression of cartilage‐degrading genes and shift the cells towards glycolysis [37, 49, 50].

Mitochondrial dysfunction in OA chondrocytes is well‐documented, characterised by altered mitochondrial structure, impaired dynamics, and respiratory function, as well as reduced biogenesis, altered calcium level modulation and increased oxidative stress, leading to chondrocyte apoptosis [51, 52, 53]. These dysregulations could account for some of the cartilage degrading processes that occur in OA, such as the inflammation‐driven breakdown of the matrix, increased oxidative stress, increased apoptosis, and the calcification of the cartilage matrix [53]. Mitochondrial dysregulation contributes to the vulnerability of chondrocytes to apoptosis, which is a key player in OA cartilage degradation [54]. Ca^2+^ homeostasis is, in part, controlled by mitochondrial calcium stores, and chondrocyte mitochondria are specialised for calcium transport and therefore play a role in the calcification of the ECM [55, 56]. Uptake and release of calcium occurs via the inositol‐1,4,5‐trisphosphate receptor and through the mitochondrial permeability transition pore which forms in response to excessive calcium accumulation, leading to a decrease in the mitochondrial membrane potential [57]. This loss of the membrane potential drives both the release of calcium and cytochrome c, resulting in apoptosis [57]. Chondrocyte apoptosis has been described to increase in tandem with the amount of cartilage damage [58]. Additionally, a depolarised mitochondrial membrane causes dysregulated PINK1‐parkin‐mediated mitophagy [59]. Impaired respiratory function is also seen in the mitochondria of OA chondrocytes. Notably, there is a reduction in the electron transport chain complexes II and III in the chondrocytes of OA patients compared with healthy controls, as well as a decrease in mitochondrial membrane potential which is required for OXPHOS‐derived ATP synthesis [60]. Furthermore, MMP expression is affected by the inhibition of complexes III and V of the mitochondrial electron transport chain, where it reduces MMP‐13 mRNA expression. Inhibition of complex V drove MMP‐1 and MMP‐3 mRNA expression [61]. Mitochondrial dysfunction triggers a perpetual cycle of oxidative stress, increased reactive oxygen species (ROS) production, and mtDNA damage, which collectively drive the progression of chronic degenerative diseases [58]. ROS accumulation in chondrocytes combined with a decrease in collagen synthesis and increased expression of MMPs and aggrecanases drive a reprogramming from an anabolic to a catabolic state in chondrocytes, leading to cartilage degradation [62].

**FIGURE 2:**
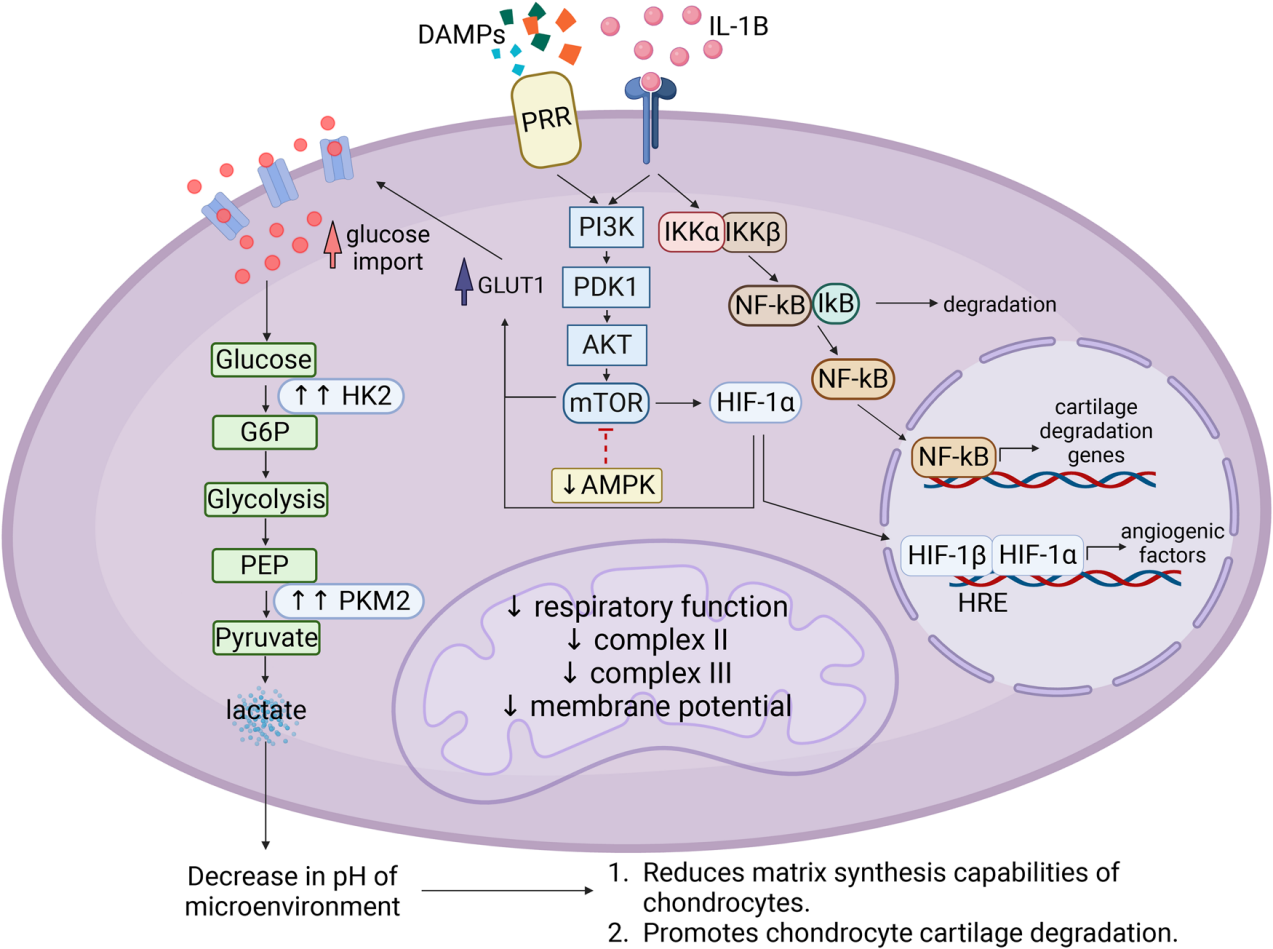
Metabolic reprogramming in osteoarthritis (OA) chondrocytes. Stimulation of chondrocytes with PAMPs, DAMPs, or cytokines such as IL‐1β collectively drives OA disease progression through multiple signalling pathways. One major pathway activated in OA chondrocytes is the PI3K/PDK1/Akt signalling cascade, leading to downstream mTOR activation, a master regulator of cell metabolism. mTOR activation enhances glucose import by upregulating GLUT1 expression, which ultimately leads to a shift towards a highly glycolytic phenotype. This glycolytic shift is further reinforced by the upregulation of rate‐limiting glycolytic enzymes, HK2 and PKM2, driving increased glycolytic flux. One key consequence of the elevated glycolysis rate is the excessive production and export of lactate which causes a decrease in the cartilage microenvironment pH. This acidification impairs chondrocyte anabolic function, reducing their ability to produce the ECM, as well as promoting cartilage degradation, a hallmark of OA pathology. Beyond its role in metabolic reprogramming, mTOR also stabilises HIF‐1α, promoting its nuclear translocation and subsequent transcription of pro‐angiogenic factors, that promote pathological angiogenesis, a feature increasingly recognised in OA, where neovascularisation invades normal cartilage, contributing further to OA disease progression. In addition to metabolic shifts, IL‐1β‐mediated activation of NF‐κB signalling plays a pivotal role in the promotion of chondrocyte catabolism. IL‐1β induces degradation of IκB, an inhibitor of NF‐κB, leading to the transcriptional upregulation of cartilage degradation genes, including MMPs and aggrecanases, driving the acceleration of ECM breakdown, and compromising cartilage integrity. Mitochondrial dysfunction is also an important feature of OA chondrocytes, and is characterised by decreased respiratory function, lower levels of ETC complex II and complex III and a drop in membrane potential. This dysfunction compromises cellular energy homeostasis and contributes to chondrocyte senescence, a state in which they adopt a pro‐inflammatory, catabolic phenotype, exacerbating joint tissue degradation. DAMPs—damage associated molecular patterns; PI3K—phosphoinositide 3‐kinase; PRR—pattern recognition receptor; IL‐1β—interleukin 1 beta; PDK1—pyruvate dehydrogenase kinase 1; AKT—protein kinase B; mTOR—mitochondrial target of rapamycin; AMPK—adenosine monophosphate‐activated protein kinase; GLUT1—glucose transporter 1; HK2—hexokinase 2; G6P—glucose 6‐phosphate; PEP—phosphoenolpyruvate; PKM2–pyruvate kinase isozymes M2; HIF‐1α—hypoxia‐inducible factor 1‐alpha; HIF‐1β—hypoxia‐inducible factor 1‐beta; HRE—hypoxia response elements; IκB—Inhibitor of Nuclear Factor Kappa B; IKKα—IκB kinase alpha; IKKβ—IκB kinase beta; NF‐κB—nuclear factor‐kappa B. Created in https://BioRender.com.

NADPH oxidase 4 through its induction of ROS, has also been implicated in modulating MMP expression and other matrix‐degrading enzymes such as disintegrins [63]. When chondrocytes are stimulated by inflammatory cytokines resulting in NF‐κB activation, a significant decrease in OXPHOS is observed, which is consistent with the observed increase in glycolysis [37, 64]. Although less studied in chondrocytes, other mitochondrial enzymes such as succinate dehydrogenase (SDH) are likely to also be involved in regulating chondrocyte function and behaviour [65]. Additionally, 4‐aminobutyrate aminotransferase (Abat), an enzyme responsible for succinate production has been implicated in driving OA, where increased succinate promotes flux through the TCA cycle and enhanced mitochondrial respiration as well as catabolic gene expression, including Runx2 and Mmp13, likely via IL‐1β [66]. Furthermore, Abat inhibition demonstrates protective effects against OA [66]. Metabolic changes in OA chondrocytes are illustrated in Figure 2, and Table 1 details a comparison of the metabolic characteristics of RA and OA.

**TABLE 1:** A comparison of the metabolic features of RA and OA.

Disease model	Cell type	Metabolic features
Rheumatoid arthritis models	FLS	mTOR activation
		Enhanced glycolysis
		Reduced OXPHOS
		HIF‐1α activation
		Lower amino acid levels
	Osteoclasts	Elevated FGF production
Osteoarthritis models	FLS	Enhanced glycolysis
		Reduced OXPHOS
		Higher amino acid levels
	Chondrocytes	mTOR activation
		Enhanced glycolysis
		Reduced OXPHOS
		Reduced AMPK activation

## 4 Macrophages Use Metabolites to Communicate With Fibroblasts and Chondrocytes

**FIGURE 3:**
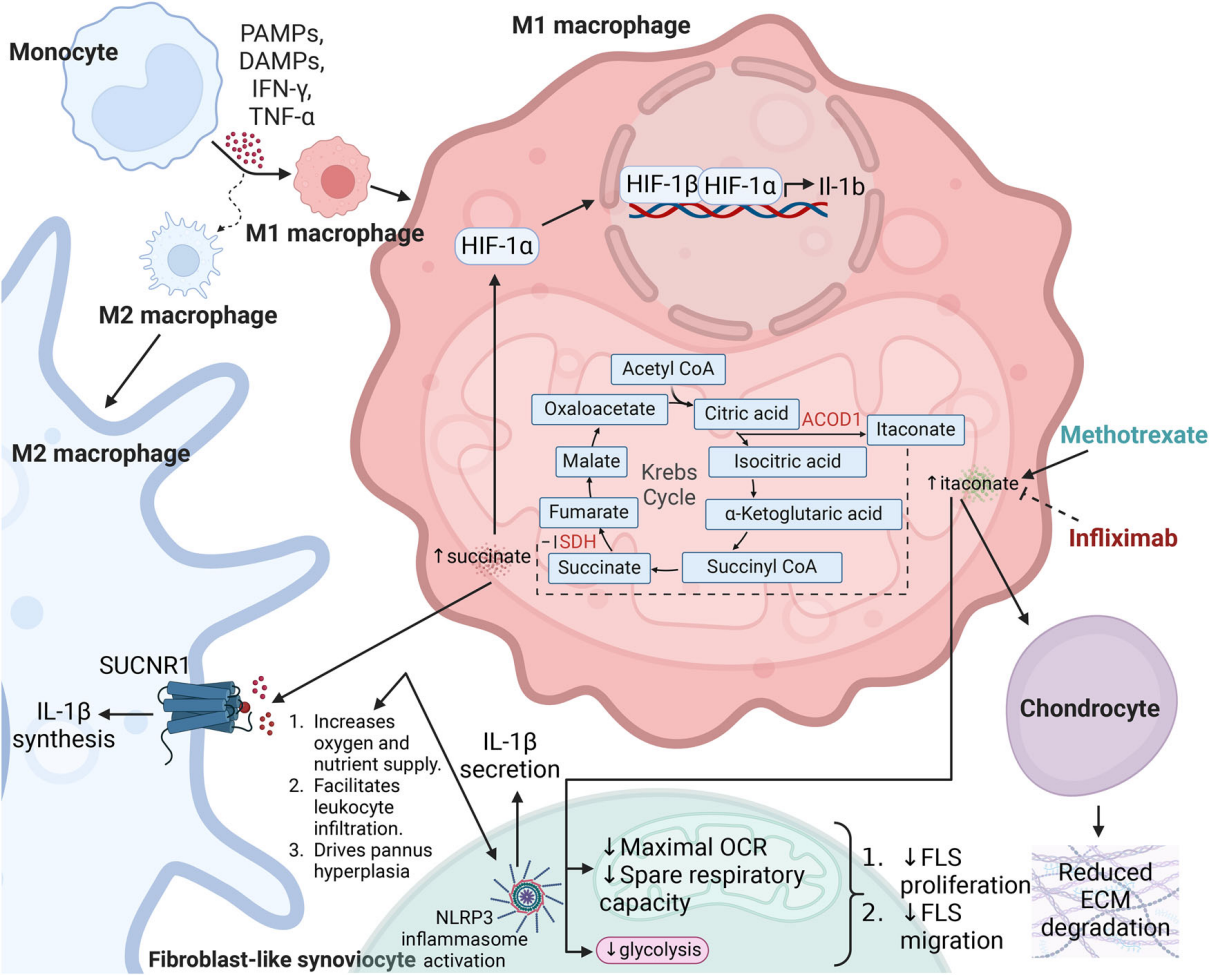
Metabolic crosstalk between macrophages, FLS and chondrocytes in arthritis. In OA, monocytes are exposed to PAMPs, DAMPs, and cytokines such as IFN‐γ and TNF‐α, driving their polarisation into classically activated (M1) macrophages, which are pro‐inflammatory and contribute to arthritis pathogenesis. A defining feature of M1 macrophages is the metabolic reprogramming of the TCA cycle, where it gets broken in two critical places [83]. First, there is an accumulation of citrate that is subsequently re‐routed for itaconate synthesis via ACOD1 and second, due to itaconate‐mediated SDH inhibition, there is a pronounced build‐up of succinate, another key inflammatory metabolite. The accumulation of succinate plays an important role in arthritis pathogenesis by acting both intracellularly and extracellularly. Succinate can stabilise HIF‐1α within the macrophage, to promote IL‐1β transcription in macrophages, while also acting in a paracrine manner by binding to SUCNR1 on M2 macrophages driving their synthesis of IL‐1β, reinforcing the inflammatory milieu. It can also drive an increase in oxygen and nutrient supply to the inflamed joint as well as promote leukocyte infiltration and pannus hyperplasia, all of which exacerbate arthritis progression. Beyond macrophages, succinate can trigger NLRP3 inflammasome activation in FLS, elevating IL‐1β levels, and perpetuating joint inflammation. In contrast to succinate's inflammatory role, itaconate exerts immunomodulatory effects by suppressing glycolysis and OXPHOS in FLS, leading to reduced proliferation and migration, which may help limit synovial hyperplasia. Moreover, itaconate signalling to chondrocytes has been linked to reduced ECM degradation. Itaconate's role in arthritis appears to be context‐dependent as revealed by pharmacological studies. Methotrexate, a commonly used DMARD increases itaconate levels whereas, infliximab, a TNF‐α inhibitor, reduces itaconate levels, suggesting a complex relationship between itaconate metabolism and inflammatory signalling in arthritis. Functionally, itaconate causes both decreased glycolysis as well as decreased maximal OCR and spare respiratory capacity in FLS. This altered FLS metabolism contributes to their reduction in proliferation and migration, ultimately restricting their invasive behaviour and thereby mitigating joint damage. Furthermore, itaconate also signals to chondrocytes, reducing their degradation of the ECM, which is a critical factor in cartilage breakdown during arthritis. This protective function of itaconate highlights its potential as a metabolic target for modulating disease progression. PAMPs—pathogen‐associated molecular patterns; DAMPs—damage‐associated molecular patterns; IFN‐γ—interferon‐gamma; TNF‐α—tumour necrosis factor‐alpha; M1 macrophage—classically activated macrophage; M2 macrophage—alternatively activated macrophage; HIF‐1α—hypoxia‐inducible factor 1‐alpha; HIF‐1β—hypoxia‐inducible factor 1‐beta; IL‐1β—interleukin 1 beta; ACOD1—aconitate decarboxylase 1; SDH—succinate dehydrogenase; SUCNR1—succinate receptor 1; NLRP3—nucleotide‐binding domain, leucine‐rich‐containing family, pyrin domain‐containing‐3; OCR—oxygen consumption rate; FLS—fibroblast‐like synoviocyte; ECM—extracellular matrix. Created in https://BioRender.com.

Metabolomics has revealed elevated levels of TCA cycle intermediates, especially succinate, in RA patient synovial fluid, hinting at its role as an important immunometabolite in the context of RA [67, 68, 69]. Inflammatory macrophages are likely to be the main sources of succinate in RA synovial fluid and succinate is the major driver of IL‐1β production in macrophages via the stabilisation of HIF‐1α, as illustrated in Figure 3 [69]. Historically, succinate has been perceived primarily as a pro‐inflammatory signal; however, evidence is building to bring it into view as more of an immunomodulatory metabolite, capable of exerting anti‐inflammatory effects in certain contexts.

Interestingly, while M1 macrophages produce succinate, the receptor SUCNR1 (also known as GPR91) is predominantly expressed in anti‐inflammatory macrophages [70, 71]. Accumulated succinate has been shown to function in stabilising HIF‐1α in macrophages, contributing to IL‐1β synthesis via SUCNR1 binding [23, 69]. Furthermore, accumulated succinate can activate the NLRP3 inflammasome in FLS in a rat arthritis model, enhancing IL‐1β secretion [72]. In murine models, succinate has been implicated in promoting angiogenesis in the synovium, increasing oxygen and nutrient supply to the inflamed joint, and facilitating leukocyte infiltration, as well as hyperplasia in the pannus [73, 74].

As outlined in Figure 3, itaconate, alongside succinate has emerged as a crucial immunometabolite in the pathogenesis of arthritis. The enzyme aconitate decarboxylase 1 (ACOD1) catalyses the conversion of cis‐aconitate to itaconate, primarily in inflammatory macrophages. Itaconate is mainly derived from macrophages and neutrophils and plays a central role in immune regulation by activating NRF2 and suppressing the NLRP3 inflammasome [75, 76]. However, its function in RA is multifaceted and context‐dependent. Elevated itaconate levels in the serum and urine of murine arthritis models are positively associated with disease activity, yet increased plasma itaconate in early RA patients has been linked to a reduction in overall disease activity, and its levels in human PBMCs were inversely correlated with disease activity [77, 78, 79]. Pharmacological interventions further underscore itaconate's complexity in RA. Methotrexate, a first‐line treatment for RA, enhances systemic itaconate levels, while in Tg197 mice, RA disease progression correlates with rising itaconate concentrations that decrease following infliximab‐mediated anti‐inflammatory therapy [78]. It is possible that the elevated itaconate is unable to counter the ongoing inflammation. Recent mechanistic insights reveal that itaconate significantly decreases basal and aerobic glycolysis along with maximal oxygen consumption rate (OCR) and spare respiratory capacity in RA FLS [80]. This study also showed that itaconate was capable of the reducing proliferation and migration of FLS in vitro [80].

IRG1 expression is significantly increased in OA patient cartilage compared with healthy controls, as well as after IL‐1β stimulation [81]. Supplementation with exogenous itaconate could effectively reverse the inflammatory response induced by IL‐1β in chondrocytes, reducing ECM degradation [82].

## 5 Therapeutic Targeting of Metabolism in RA and OA

HIF‐1α in combination with PKM2, binds to the IL‐1β promoter, increasing its production [84]. Evidence suggests that HIF‐1α knockdown decreases the expression of PFKP and LDHA, while upregulating enzymes involved in OXPHOS and fatty acid oxidation, indicating that targeting HIF‐1α could effectively shift the metabolic balance from glycolysis back to OXPHOS, thereby reducing RA FLS proliferation [85]. As detailed in Table 2, the inhibition of this pathway by compounds such as Taxol shows promise in alleviating RA. The chemotherapeutic agent Taxol, known to inhibit HIF‐1α production, has been demonstrated to mitigate collagen‐induced arthritis through modulation of the HIF‐1α/VEGF/ANG‐1 axis [86]. Moreover, the conformation of HIF‐1 relies on the presence of heat shock protein 90 (HSP90), suggesting that HSP90 inhibition could diminish HIF‐1 activity, thereby decreasing glycolysis and presenting a potential therapeutic strategy for RA. The HSP90 inhibitor, geldanamycin through the MAPK and NF‐κB pathways, has been shown to stop TNF‐α‐driven FLS proliferation and enhance apoptosis [87]. A key limitation of these current studies is the potential for off‐target effects, as many inhibitors, including Taxol and HSP90 inhibitors, affect multiple signalling pathways. Hypersensitivity, neutropenia, neurotoxicity, mucositis, and cardiac toxicity have been reported with Taxol use [88]. Additionally, geldanamycin has been shown to affect neurite outgrowth by targeting microtubule‐binding proteins and kinases, leading to neuronal impairment [89].

**TABLE 2:** Therapeutic approaches that target metabolism in RA and OA.

Disease model	Therapeutic	Mechanism of action
Rheumatoid arthritis models	PKM2 inhibitor (e.g., Shikonin)	Suppresses glycolytic enzymes via the PI3K/Akt/mTOR axis in FLS.
	HIF‐1α inhibitor (e.g., Taxol)	Re‐establishes metabolic balance via the upregulation of the enzymes involved in OXPHOS and fatty acid oxidation. Causes G2/M phase cell cycle arrest and apoptosis in RA FLS [129].
	HSP90 inhibitor (e.g., geldanamycin)	Diminishes HIF‐1 activity, decreasing glycolysis. Geldanamycin stops TNF‐α‐driven FLS proliferation and enhances apoptosis.
	4‐Octyl itaconate (4‐OI)	Reduces ROS production. Limits pro‐inflammatory cytokine production.
	Dimethyl malonate (DMM)	Competitive inhibitor of SDH. Inhibit the HIF‐1α/VEGF axis in FLS, limiting succinate accumulation, and blocking angiogenesis.
	SUCNR1 blockade	Deficiency of this receptor has been shown to attenuate antigen‐induced arthritis.
	mTOR inhibitor (e.g., rapamycin)	Activation of autophagy and inhibition of VEGF, collagen type X α1 chain, and MMP13.
Osteoarthritis Models	GLUT1 inhibitor (e.g., BAY‐876)	Reduced glucose import, resulting in reduced glycolysis in FLS.
	PKM2 inhibitor	Prevents OA chondrocyte proliferation.
	AMPK activator	Suppress NF‐κB activation in chondrocytes. Reduce chondrocyte oxidative stress. Dampen inflammatory and catabolic processes.
	PGC‐1α	Promote mitochondrial biogenesis in chondrocytes. Regulate mitophagy in chondrocytes. Reduce chondrocyte oxidative stress. Promotes chondrocyte mtDNA replication and expression.
	Inhibiting the mitochondrial pyruvate carrier	Reduce mitochondrial respiration. Reduce ROS production.
	Antioxidants, e.g., melatonin, dihydromyricetin, quercetin, taurine, and diallyl disulfide	Reduce mitochondrial dysfunction. Avoid excessive chondrocyte apoptosis.
	4‐Octyl itaconate (4‐OI)	Activation of Nrf2 inhibits cartilage destruction. Reverses IL‐1β‐induced autophagy by inhibiting the PI3K/AKT/mTOR pathway in chondrocytes. Mitigates ROS production and maintains GPX4 expression, reducing the damaging effects of IL‐1β and erastin on chondrocytes by inhibiting ferroptosis.

Front‐line RA therapies including glucocorticoids and DMARDs such as methotrexate, leflunomide, and hydroxychloroquine modify metabolism, affecting the glycolytic and mTOR pathways, highlighting the great potential that targeting metabolites holds for the treatment of RA [90]. Interestingly, glucocorticoids (GCs), mainstays in RA management, may operate through the remodelling of mitochondrial metabolism, facilitating the production of itaconate, which limits the inflammatory response by minimising pro‐inflammatory cytokine production [91]. While GCs are known for their anti‐inflammatory actions, such as blocking NF‐κB and AP‐1 family members, the underlying mechanisms remain incompletely understood [91]. This recent study demonstrates that GCs prevent LPS‐induced lung inflammation in wild‐type mice, but fail to do so in Acod1^−/−^ mice, highlighting the dependency of GC activity on ACOD1‐mediated itaconate production. Their cross‐sectional analysis also identified increased serum itaconate levels in RA patients receiving GCs compared with those not on GC therapy. Daily GC administration in the K/BxN serum transfer arthritis mouse model similarly increased serum itaconate levels and significantly reduced arthritis severity. However, in the absence of ACOD1, GCs were unable to alter the disease course, further emphasising the critical role of ACOD1 in mediating the anti‐inflammatory effects of GCs. Carbon tracing experiments using uniformly labelled (U)‐[13C] glucose revealed that GCs redirect glucose metabolism of proinflammatory macrophages, increasing pyruvate entry into the TCA cycle. Notably, blocking the TCA cycle through HIF‐1α or inhibiting mitochondrial pyruvate import with UK5099 in LPS‐activated macrophages reduced the ability of GCs to exert their anti‐inflammatory effects. Interestingly, GCs did not directly act via ACOD1, the enzyme responsible for itaconate synthesis, but rather by maintaining the availability of aconitate, the ACOD1 substrate. This underscores the potential significance of metabolites such as itaconate for RA therapy. However, the reliance of GC activity on ACOD1 raises important questions about variability in metabolic enzyme expression among individual RA patients and how this might influence treatment outcomes. This highlights the importance of work on the administration of the derivative of itaconate, 4‐OI which has anti‐inflammatory effects in many models of inflammation [92, 93]. 4‐OI has shown great promise, as it reduces ROS production, limits pro‐inflammatory cytokine synthesis, and prevents cartilage degradation. Given that prolonged usage of GCs is associated with adverse effects such as osteoporosis, cardiovascular, and neuropsychiatric issues as well as metabolic dysfunction, further work is required to assess whether ACOD1 modulation or itaconate could provide a safer alternative therapeutic avenue [94].

Due to its pro‐inflammatory characteristics, succinate could also be a promising metabolite target for therapeutics. Thus, the administration of dimethyl malonate (DMM), a cell‐permeable prodrug of malonate and competitive inhibitor of SDH, may hold therapeutic potential for RA where conditions are hypoxic. DMM has been demonstrated to inhibit the HIF‐1α/VEGF axis in hypoxia‐treated FLS, ultimately blocking angiogenesis [73]. Additionally, SUCNR1, being a GPCR presents a potential target for therapeutic intervention. Notably, deficiency of this receptor has been shown to attenuate antigen‐induced arthritis [95]. SUCNR1 antagonists have been proposed as a novel treatment approach for RA, which has been shown to block the secretion of IL‐1β from U937 cells that have been stimulated with human synovial fluid or succinate [69]. Longitudinal clinical trials will be essential to establishing whether succinate‐targeting therapies can induce sustained arthritis remission or if their efficacy declines with time due to metabolic adaptation and compensatory mechanisms. The therapeutic effects of other succinate‐related strategies are described in Table 1.

In RA, OCs are also involved in disease pathogenesis. Itaconate may also be interesting therapeutically via targeting of OCs. A recent study has shown that during OC differentiation, the TCA cycle of pre‐OCs becomes profoundly rewired, where the gene *Irg1* is induced, causing subsequent itaconate synthesis via the Acod1 enzyme [96]. Irg1^−/−^ OCs have shown enhanced aerobic glycolysis and elevated numbers of OCs in vitro and in vivo, with the deficiency of Acod1 being associated with exacerbated inflammation as well as elevated bone degradation [79]. In addition to the Irg1/itaconate axis being shown to suppress OC differentiation, it was also found important for stimulating osteoblasts in mice and humans [96]. Arthritis development in the K/BxN serum transfer model is accompanied by much osteoblast‐derived formation of new bone, and this new bone formation was found to be deficient in Irg1^−/−^ mice compared with Irg1^+/+^ mice, indicating a role for Acod1 in the formation of this new bone. The authors demonstrated elevated expression of transcription factors involved in osteogenic differentiation and genes encoding for ECM proteins upon itaconate stimulation. Interestingly, when pre‐osteoblasts start the enter the differentiation phase, they have been shown to stop proliferating and subsequently increase their glycolytic activity [97, 98, 99]. This same effect was also seen with the addition of itaconate [96]. It would be important for future studies to investigate whether targeting the Irg1/itaconate axis could be integrated with existing therapies to enhance bone protection in RA. This has the potential to give a synergistic effect by addressing both the inflammatory and metabolic dysfunction in the joint microenvironment. Understanding the effect of itaconate not only on OC activity but also on the differentiation of osteoblasts in the context of RA could provide a potential dual‐target approach for promoting bone homeostasis while simultaneously controlling inflammation.

Multiple metabolic interventions can be utilised to address OA pathogenesis, with several targets, such as 4‐OI and PKM2 inhibitors, demonstrating therapeutic potential across both RA and OA models.

Blocking AMPK and mTOR is being investigated in the context of OA. Reduced articular cartilage damage was observed in mice with OA that were administered injections of rapamycin, an mTOR inhibitor. This was found to be caused by the activation of autophagy and the inhibition of VEGF, collagen type X α1 chain, and MMP13 [100]. While mTOR inhibitors could help mitigate OA‐causing mechanisms, chronic inhibition could impair normal physiological processes in non‐target tissues, resulting in many adverse side effects, including renal issues, dysfunctional wound healing, metabolic disorders, endocrine disorders and others [101].

Glycolytic metabolism also emerges as a critical therapeutic target in OA. The low‐grade inflammation characteristic of OA can induce hypoxia, driving an increase in glycolysis [36, 37, 38]. It is well established that chondrocyte hypertrophy and the degradation of the ECM which contribute to OA development can be caused by glucose metabolism disorders such as diabetes mellitus [102, 103]. Growing evidence indicates that the metabolic switch to glycolysis allows cells to generate ATP, playing a crucial role in triggering inflammatory processes in OA [104]. Consequently, re‐establishing the balance between glycolysis and OXPHOS may be useful in the treatment of OA, perhaps via targeting the glucose transporters or glycolytic enzymes.

The initial step in glycolysis involves chondrocyte uptake of glucose via GLUT1, which is notably upregulated in hypoxic conditions [105]. When GLUT1 is consistently upregulated, cartilage becomes degraded [41]. Inhibition of GLUT1 has been shown to curtail tumour cell growth [106, 107]. Currently, the GLUT1 inhibitor with the most translational potential is BAY‐876, showing effective anti‐cancer activity [108, 109]. This data suggests potential applications in decreasing OA FLS proliferation. However, it should be emphasised that GLUT1 is widely expressed across various cell types, so blocking its activity may lead to unintended side effects [110]. For instance, human erythrocytes express the highest levels of GLUT1 per cell, where it also facilitates L‐dehydroascorbic acid transport [111]. Additionally, endothelial cells of the blood–brain barrier express GLUT1 for glucose transport from blood to the CNS. Unsurprisingly, neurological symptoms are present in both patients with GLUT1 dysfunction as well as in patients undergoing pharmacological inhibition of GLUT1 [110]. Interestingly, with GLUT1 dysfunction, most of these symptoms occur during childhood and adolescence and lessen or attenuate in adulthood, indicating that the use of this kind of treatment should be limited to adolescent and adult patients and only be used briefly [110]. PKM2 is another glycolytic enzyme upregulated in OA chondrocytes. Studies have shown that PKM2 inhibition prevents OA chondrocyte proliferation, further establishing its potential as an OA therapeutic target [102].

Targeting chondrocyte mitochondrial pathways offers a promising strategy to counteract mitochondrial dysfunction and mitigate OA. When AMPK is activated, it can promote the suppression of ATP‐consuming processes while stimulating ATP‐generating pathways [112, 113]. In addition, chondrocyte AMPK activation has been shown to dampen NF‐κB activation as well as oxidative stress, and inflammatory and catabolic processes [114]. AMPK and SIRT1/3 modulate the activity of each other [115]. It is becoming clearer that SIRT1 is involved in driving mitochondrial dysfunction and OA disease progression [116]. Autophagy is suppressed in OA, and SIRT1 is known to induce autophagy [117]. Since the promotion of autophagy is protective for chondrocytes, targeting AMPK or SIRT1 therapeutically could be beneficial to OA disease mitigation [52, 118, 119]. Peroxisome proliferator‐activated receptor γ coactivator 1α (PGC‐1α) provides another potential therapeutic target in OA as the blockade of catabolic processes and dampening of oxidative stress by AMPK in chondrocytes requires PGC‐1α [120]. PGC‐1α can slow OA progression via modulation of chondrocyte mitochondrial dynamics such as their biogenesis via NRF2, NRF1 and TFAM, as well as limiting oxidative stress, mitophagy and mtDNA replication (121]. Furthermore, it has been shown that mitochondrial respiration as well as ROS production can be reduced by inhibiting the mitochondrial pyruvate carrier, highlighting it as a potential OA therapeutic strategy [54]. Targeting mitochondrial metabolism has also been studied in relation to OA, specifically with nicotinamide riboside and pyrroloquinoline quinone being protective in OA [122, 123]. Exogenous drugs are another potential treatment avenue for OA and could be used to therapeutically target mitochondria. Antioxidants such as melatonin, dihydromyricetin, quercetin, taurine, and diallyl disulfide have been suggested as potential OA drugs due to their ability to reduce mitochondrial dysfunction and avoid excessive chondrocyte apoptosis, which is reviewed in detail elsewhere [124].

Similar to RA, there is a strong body of work supporting the therapeutic role of 4‐OI in OA. Activation of Nrf2 has been shown to inhibit cartilage destruction [125, 126]. A new investigation found that 4‐OI can mitigate inflammation, ECM degradation and chondrocyte senescence induced by IL‐1β through the activation of Nrf2, which in turn inhibits the STING‐dependent NF‐κB pathway [82]. Another study demonstrated that 4‐OI can reverse IL‐1β‐induced autophagy by inhibiting the PI3K/Akt/mTOR pathway, leading to reduced cartilage degradation in rat models of OA [127]. A recent study demonstrated that 4‐OI can mitigate ROS production and maintain glutathione peroxidase 4 (GPX4) expression, effectively countering the damaging effects of IL‐1β and erastin on chondrocytes by inhibiting ferroptosis [128]. This ultimately alleviated the observed IL‐1β‐induced chondrocyte activation.

## 6 Conclusion

Metabolic reprogramming of both immune cells and stromal cells underpins the pathology of RA and OA and hence presents a promising frontier for therapeutic intervention. Novel approaches that target metabolites or exploit them in the case of itaconate could prove useful in the halting of joint destruction in OA or perhaps in conjunction with biologics such as anti‐TNF in RA. Further research is necessary to unravel the intricate metabolic networks and their implication in joint diseases, and a deeper understanding of these could pave the way for novel therapeutics.

## Author Contributions

Órlaith C. Henry and Luke A. J. O'Neill contributed equally to all aspects of this study.

## Ethics Statement

No experiments were conducted with animals for this manuscript, and this manuscript does not contain human studies.

## Conflicts of Interest

LON is a consultant for Sitryx and has no other competing interests to declare. OH declares no conflicts of interest.

### Peer Review

The peer review history for this article is available at https://publons.com/publon/10.1002/eji.202451381.

## Data Availability

Data sharing is not applicable to this article as no datasets were generated or analysed during the current study.
